# Study on the preventive effect of dexmedetomidine on anesthetic associated sleep disturbance in young to middle-aged female patients undergoing hysteroscopy: a study protocol for a crossover randomized controlled trial

**DOI:** 10.1186/s13063-024-08311-6

**Published:** 2024-07-15

**Authors:** Xueru Li, Lijuan Yan, Linhong Wang, Hanshen Chen, Bin Yang

**Affiliations:** 1 Department of Anesthesiology, The First Affiliated Hospital of Xiamen University, Fujian, 361000 China; 2 Department of Anesthesiology, The First Affiliated Hospital of Fujian Medical University, Fujian, 350005 China; 3 School of Clinical Medicine, Fujian Medical University, Fujian, 350000 China

**Keywords:** Sleep disturbance, Propofol, Dexmedetomidine, Crossover randomized controlled trial

## Abstract

**Background:**

Postoperative sleep disturbance has a potentially detrimental effect on postoperative recovery. Perioperative patients are affected by several factors. General anesthesia induces a non-physiological state that does not resemble natural sleep. Exposure to propofol/sevoflurane can lead to desynchronization of the circadian rhythm, which may result in postoperative sleep disturbance characterized by mid-cycle advancement of sleep and daytime sleepiness. Dexmedetomidine is a highly selective α2-adrenoceptor agonist with a unique sedative effect that facilitates the transition from sleep to wakefulness. Basic research has shown that dexmedetomidine induces deep sedation, similar to physical sleep, and helps maintain forebrain connectivity, which is likely to reduce delirium after surgery. The aim of this study is to evaluate the influence of exposure to the mono-anesthetic propofol on the development of postoperative sleep disturbance in young and middle-aged female patients undergoing hysteroscopy and whether prophylactic administration of dexmedetomidine influences reducing postoperative sleep disturbance.

**Methods:**

This prospective randomized controlled trial (RCT) will include 150 patients undergoing hysteroscopy at the First Affiliated Hospital of Xiamen University. Participants will be randomly assigned to three groups in a 1:1:1 ratio. The dexmedetomidine group will have two subgroups and will receive a nasal spray of 0.2 µg/kg or 0.5 µg/kg 25 min before surgery, while the control group will receive a saline nasal spray. Three groups will undergo hysteroscopy with propofol-based TIVA according to the same scheme. Sleep quality will be measured using a wearable device and double-blind sleep assessments will be performed before surgery and 1, 3, and 7 days after surgery. SPSS 2.0 is used for statistical analysis. A χ^2^ test is used to compare groups, and *t*-test is used to determine statistical the significance of continuous variables.

**Discussion:**

The purpose of this study is to investigate the incidence of propofol-associated sleep disorders and to test a combination of dexmedetomidine anesthesia regimen for the prevention of postoperative sleep disorders. This study will help to improve patients’ postoperative satisfaction and provide a new strategy for comfortable perioperative medical treatment.

**Trial registration:**

ClinicalTrials.gov NCT06281561. Registered on February 24, 2024.

## Introduction

### Background and rationale {6a}

General anesthesia induces a drug-dependent state of unconsciousness and disrupts the existence of repeated 90-min cycles of non-REM and REM sleep (only non-REM), which significantly suppresses normal physiological sleep in a given postoperative period [1]. Previous studies have reported that it has been demonstrated that these postoperative sleep disturbances are associated with an increased incidence of complications, including daytime fatigue, anxiety, pain, and a significant impact on mental and cognitive function [2]. Furthermore, sleep disturbance after general anesthesia in the elderly has been shown to increase the incidence of acute postoperative delirium and long-term cognitive dysfunction. Some patients undergoing gastroenterostomy experience sleep disturbances up to 7 days after being exposed to propofol for only a few minutes [3, 4]. The precise impact of anesthetic on postoperative sleep disturbance remains unclear. There is a higher prevalence of sleep disorders in female patients compared to males [5], suggesting that comprehensive investigations in this cohort may identify a potential intrinsic association between anesthetics and sleep disturbances.

Dexmedetomidine is a potent, highly selective α2-adrenoceptor agonist with sedative, analgesic, anxiolytic, sympatholytic, and opioid-sparing properties. Dexmedetomidine induces a unique sedative response with an easy transition from sleep to wakefulness, allowing the patient to be cooperative and communicative when stimulated [6]. Basic research has shown that dexmedetomidine induces deep sedation similar to physical sleep and helps maintain forebrain connectivity, which is likely to result in less delirium after surgery [7]. Compared with propofol anesthesia, saliency networks and thalamic connectivity with key nodes of arousal were relatively preserved in dexmedetomidine-induced unresponsiveness, which was similar to N3 physical sleep [8, 9]. This has been demonstrated to be potentially beneficial in restoring sleep disturbance in the early stage of hepatic encephalopathy [10]. Furthermore, clinical trials have shown that dexmedetomidine-soaked nasal packing can improve sleep quality after nasal endoscopic surgery [11]. Recent studies have indicated that dexmedetomidine may regulate sleep–wake cycle abnormalities in the early stages of injury associated with surgical trauma [12]. Nevertheless, few studies have evaluated the effects of general anesthesia as an independent factor (timing and concentration) on postoperative sleep quality and recovery time, and the effects of prophylactic dexmedetomidine administration on anesthesia-associated sleep disturbances have not been elucidated.

## Objectives {7}

The objective of this study is to investigate the incidence of propofol-associated sleep disturbance in young and middle-aged women and to evaluate the efficacy of a combined anesthetic protocol in preventing postoperative sleep disturbances.

## Trial design {8}

**Fig. 1:**
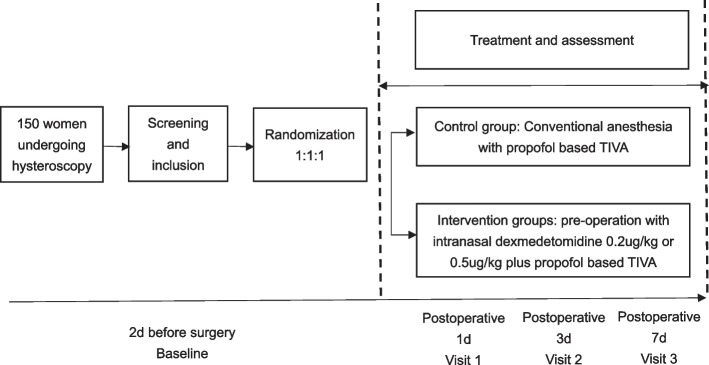
Study outline. TIVA, total intravenous anesthesia. The first assessment was conducted in the anesthesia clinic and after surgery through on-site or telephone visits

This is a randomized, controlled trial in female patients undergoing propofol-based anesthesia regimen. A flowchart of the study design is presented in Fig. 1. Prior to the commencement of the study, informed consent will be obtained from patients at the anesthetic clinic and subsequently assessed by a senior anesthesiologist. Recruited patients will be assigned wearable devices that monitored their objective sleep quality for nine consecutive days, from 2 days before general anesthesia to 7 days after surgery. Before the experiment, they are randomly divided into three groups, which are given dexmedetomidine 0.5 mg/kg, 0.2 mg/kg, and the same amount of normal saline, respectively, to observe the postoperative situation. Subjective sleep quality is determined according to the Pittsburgh Sleep Quality Index (PSQI) and Athens Insomnia Scale (AIS) completed by the patients, while anxiety and depression is assessed according to the Hospital Anxiety and Depression Scale (HADS) completed by the patients after surgery.

## Methods: participants, interventions, and outcomes

### Study setting {9}

This RCT will be conducted in the First Affiliated Hospital of Xiamen University, China.

### Eligibility criteria {10}

Inclusion criteria:

1. Patients undergoing hysteroscopy in the hospital.
2. Age must be at least 23 years old.
3. American Society of Anesthesiologists Classification (ASA) I–II.
4. No significant cardiovascular disease, liver, or kidney function within normal limits.
5. Fully informed about the study and willing to participate by signing the informed consent form.

Exclusion criteria:

1. ASA III–IV, severe cardiovascular disease, or poor physical condition.
2. A history of mental illness.
3. A history of sedative or antipsychotic drug use (neuroinhibitors, anxiolytics, antidepressants, benzodiazepines) for any reason.
4. A history of sleep disorders or night shifts.
5. Surgical complications (bleeding, reoperation, meningitis).
6. Nasal deformity or nasal trauma.

### Who will take informed consent? {26a}

Prior to undergoing hysteroscopy, all patients are interviewed by a trained anesthetist in the anesthetic clinic. At the end of the visit, patients will be given general information and will be asked to sign an informed consent form if they decide to participate.

### Additional consent provisions for collection and use of participant data and biological specimens {26b}

No human biological specimens will be collected at all. During the experiment, participants’ questionnaires and monitoring data will be stored on a secure server by the lead investigator, and seeking such informed consent is a condition of participation.

## Interventions

### Explanation for the choice of comparators {6b}

The comparator will be normal saline (NaCl 0.9%). To meet the requirement for blinding, it is not possible to use a blank control. Instead, saline is selected as placebo comparator. Saline is a transparent liquid that can also be employed to rinse the nasal passages, thereby ensuring the utmost safety for patients.

### Intervention description {11a}

In this study, all subjects meeting the inclusion criteria will be randomly assigned to one of three groups. The study will include three groups: DEX 0.2 group (0.2 µg/kg dexmedetomidine), DEX 0.5 group (0.5 µg/kg dexmedetomidine), and control group (equivalent volume of normal saline). All dexmedetomidine solutions will be diluted to 0.6 ml at different doses and prepared by a senior nurse who is not involved in the follow-up. All patients will receive no preoperative medication. On arrival in the waiting room, all patients will be examined and receive an intranasal spray of a total volume of 0.6 ml 25 min before anesthesia. Patients, anesthetists, gynecologists, and nurses are all blinded to group allocation. Patients receive a 20G intravenous cannula with lactated Ringer’s solution. Heart rate, non-invasive blood pressure, electrocardiogram, and SpO2 and BIS are monitored during hysteroscopic procedures. Propofol is administered via the TCI infusion system using the Marsh pharmacokinetic parameters with a target plasma concentration of 3.2 ng/ml in three groups. The initial starting dose of propofol is based on the results of our pre-test and BIS. If the target was achieved and the BIS was < 50, remifentanil is given at 1 µg/kg by pump and LMA is then placed. During the hysteroscopy, TCI regulates according to the Dixon up and down method, which is firstly described in 1965. TCI will be upregulated by 0.1–0.2 when patients have a somatic response (unintended movement of the angulus oris or limbs) during cervical dilation. In the absence of limb movement, the subsequent target plasma concentration is reduced by 0.1 ng/ml. Emergency equipment and drugs are available at all times. In the PACU, pain scores are assessed by a trained anesthesia nurse who is blinded to group allocation. The Numerical Rating Scale (NRS) is used every 10 min immediately after extubation. For scores of 3 or more, flurbiprofen 50 mg is administered until the patient is relieved.

### Criteria for discontinuing or modifying allocated interventions {11b}

The intervention can be discontinued at the participants’ request or following the occurrence of a related serious adverse event.

### Strategies to improve adherence to interventions {11c}

To improve patient compliance, our researchers will supervise the implementation of the intraoperative protocol during the study. There is a fixed follow-up staff after the operation. Patients who are discharged early will be followed up by telephone.

### Relevant concomitant care permitted or prohibited during the trial {11d}

No concomitant care is permitted during this trial.

### Provisions for post-trial care {30}

If participants suffer any harm from the trial, they will be treated according to the standard medical procedures.

### Outcomes {12}

The primary outcome is the incidence of PSD, which is evaluated using the PSQI and AIS on postoperative days (POD) 1, 3, and 7. Postoperative sleep disturbance is defined as having PSQI score higher than 11 or AIS score of 6 points or higher, indicating that sleep is repeatedly interrupted throughout the night, or even worse. The score below 70 measured by smartwatches will be thought of as a screening complementary indicator. The secondary outcomes included postoperative pain scores at rest and on movement at 24 h (using VAS), postoperative remedial analgesic and sedative-hypnotics consumption within 24 h, and anxiety and depression scores on POD 1, 3, and 7 (using HADS).

Postoperative complications, including nausea and vomiting, dizziness, itching, and nightmares, are noted and treated accordingly. In addition, surgery and anesthesia duration, consumption of intraoperative remifentanil, estimated infusion volume, blood loss and urine output, laryngeal mask removal time (the time from the end of surgery to airway removal of laryngeal mask), and length of stay in the post-anesthesia care unit are also recorded.

### Participant timeline {13}

See Table 1.

**Table 1:**
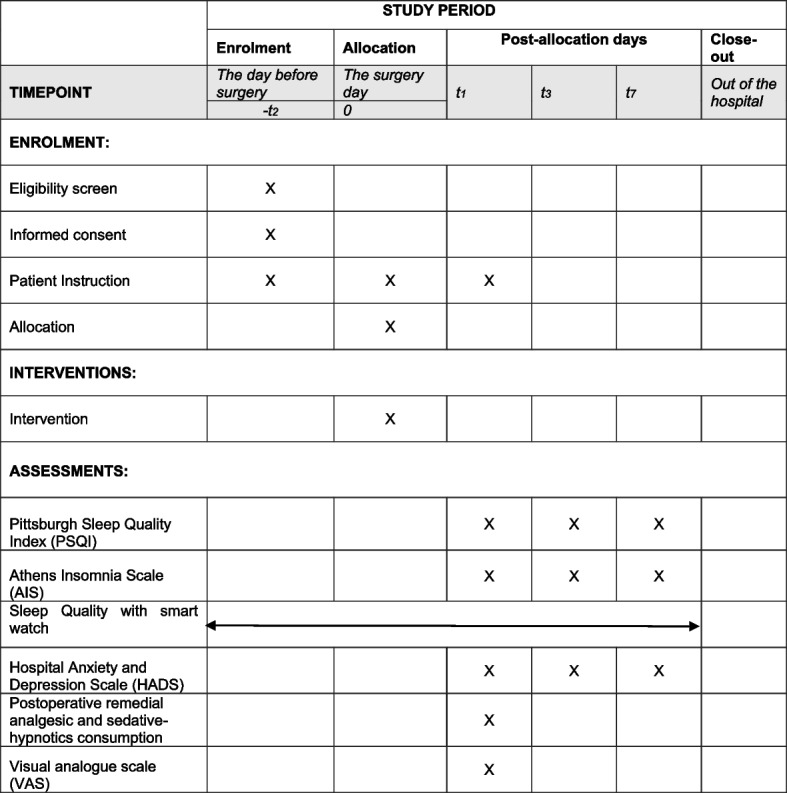
Schedule of enrolment, interventions, and assessments

### Sample size {14}

Based on the incidence of POD in patients undergoing gynecological surgery was up to 31.4% [12], the sample size of 150 participants (50 per group) was determined using G*Power software (version 3.1.9.2) based on the input parameters of a power of 0.80, an *α* level of 0.05, and repeated-measures analyses of variance (ANOVAs) with within-between interaction statistical tests. A 10% withdrawal and loss to follow-up has been taken into account.

### Recruitment {15}

All patients undergoing hysteroscopy at the First Affiliated Hospital of Xiamen University are evaluated by senior anesthesiologist in the pre-anesthesia clinic. If the patient meets the criteria, he will be fully informed with the clinical trial and be offered the possibility to enroll in the study. Recruitment is expected to be completed in 3 to 6 months.

## Assignment of interventions: allocation

### Sequence generation {16a}

Patients will be randomized via a remote web-based randomization system (OxMar Software, Oxford Minimization and Randomization, an open-source software) [13], which ensures a concealed allocation sequence. The participants will have the appointments and controls as in our usual clinical practice.

### Concealment mechanism {16b}

In accordance with the principle of blinding, an equal quantity of saline is employed for the control group, given that all fluids are colorless. A nurse who is not involved in the study will load the study drugs into the same 1-ml syringe according to the groupings and the information is contained in sequentially numbered sealed envelopes.

### Implementation {16c}

The computer will generate the allocation sequence. A senior anesthesiologist in anesthesia clinic will screen and recruit eligible patients. Blinded nurses will participate in the intervention and control drug allocation.

## Assignment of interventions: blinding

### Who will be blinded {17a}

All anesthetists and other study participants, as well as the patients, are blinded to the group allocation.

### Procedure for unblinding if needed {17b}

In the event of a medical emergency, it may be necessary to remove the blinding proper treatment. This decision must be endorsed by the attending physician and recorded in the protocol, with the utmost consideration given to minimizing bias and ensuring data quality.

## Data collection and management

### Plans for assessment and collection of outcomes {18a}

An independent advanced nurse will follow the patients for postoperative pain and complications. They also make assessments based on several measurement forms (PSQI, AIS, HADS) filled out by patients.

The PSQI score ranges from 0 to 21, wherein 0–5 represents excellent or good sleep and 16–21 represents the inability to fall asleep all night [14]. The AIS is a psychometric questionnaire to quantify sleep difficulties according to the International Statistical Classification of Diseases and Related Health Problems, Tenth Revision criteria. The AIS consists of 8 items: waking up at night, sleep induction, final awakening, total sleep duration, sleep quality, well-being, functional ability, and daytime sleepiness. The AIS score ranges from 0 to 24 points, and a total score of 6 points or higher indicates a diagnosis of insomnia [15]. The VAS score ranges from 0 (indicating no pain) to 10 (indicating intolerable pain) points [16]. The HADS consists of 14 questions, with 7 items each for the anxiety and depression subscales. The score for each item ranges from 0 to 3 points, and scores are summed to yield a separate score for anxiety (HADS-A) and depression (HADS-D). Scores of 8 points or higher are diagnosed as depression or anxiety [17].

Data for all items are recorded in the medical record and allocated to the researcher. The principal investigator or co-investigator approve each patient’s recorded observation and assessment data after double-checking the content. If there is any doubt about the data, the researcher will contacted. In this study, the monitoring will be carried out in accordance with the study plan and the procedures will be monitored continuously to ensure the research process.

### Plans to promote participant retention and complete follow-up {18b}

Patients will be fully informed of information and follow-up times. A researcher will be sent to visit the patient regularly. If the patient is discharged early, a telephone follow-up will be conducted. If intermediate patient follow-up data is missing, labeling is required.

### Data management {19}

All the data will be collected and sent to the principal investigator, who will store it online on a secure server (Baidu Cloud Disk, https://pan.baidu.com/). Only the principal investigator will have access to all the data. If other researchers wish to obtain data pertinent to this study, they are invited to the PI by email once the study has been completed.

### Confidentiality {27}

This trial will follow the Personal Information Protection Law of China. The database will be kept confidential and anonymous. It records the specific codes of patients rather than their full names. In presentations or publications arising from this study, information will be provided in such a way that patients cannot be identified. No study details can be disclosed to unauthorized third parties without prior approval.

### Plans for collection, laboratory evaluation, and storage of biological specimens for genetic or molecular analysis in this trial/future use {33}

Not applicable. No human specimens will be collected.

## Statistical methods

### Statistical methods for primary and secondary outcomes {20a}

SPSS software (version 23.0. Chicago, IL, USA) will be used for statistical analysis. The Kolmogorov–Smirnov test will be used to assess the distribution of variables. Quantitative data will be expressed as mean ± SD or median and interquartile range, while categorical data will be expressed as percentages (%). Independent *z*-tests will be employed to assess the statistical significance of differences in continuous variables, while Fisher exact tests or χ^2^ tests will be used to compare patient characteristics. VAS scores will be evaluated using the Mann–Whitney test and the χ^2^ test for categorical variables. The difference will be considered statistically significant (*P* < 0.05).

### Interim analyses {21b}

The lead researcher will submit an interim report every 12 months, which is reviewed annually by the Scientific Research Department of the First Affiliated Hospital of Xiamen University. The review process will be independent of investigators and sponsors.

### Methods for additional analyses (e.g., subgroup analyses) {20b}

Descriptive statistics will be provided for safety data. The number of patients reporting any AEs or supposed serious adverse events (SAEs) and the occurrence of specific SAEs will be tabulated.

### Methods in analysis to handle protocol non-adherence and any statistical methods to handle missing data {20c}

In the event of participants dropping out during the intervention period, they will not be excluded due to the intention-to-treat principle. Furthermore, in instances where data is missing, the continuation of observations (LOCF) method will be employed, with the last recorded observation prior to the data gap serving as a substitute.

### Plans to give access to the full protocol, participant-level data, and statistical code {31c}

The plans of this study are available on the clinical trial registration website.

## Oversight and monitoring

### Composition of the coordinating center and trial steering committee {5d}

The coordinating center consists of clinicians from the Department of Anesthesiology of the First Affiliated Hospital of Xiamen University. The principal investigators will supervise this trial and be responsible for the medical responsibility of patients. The data manager will collect the data carefully. The study coordinator will register this trial and coordinate study visits and safety reporting. There is no trial steering committee (TSC) for this trial.

### Composition of the data monitoring committee, its role and reporting structure {21a}

The safety of the trial participants will be ensured by the institutional review board and the study team as they monitor the ethical conduct of this study, ensuring that the trial is implemented according to the protocol and that data are collected appropriately.

A data monitoring committee is not required, because dexmedetomidine is routinely administered for anesthesia and the whole process is supervised by anesthetists and nurses.

### Adverse event reporting and harms {22}

This pharmacological intervention has already been used in clinical practice. And it is done under the supervision of an anesthetist and a nurse in the post-anesthesia unit. Any emergency situation will be dealt with promptly.

### Frequency and plans for auditing trial conduct {23}

The researchers (HS. Chen and B. Yang) will regularly analyze the data to make adjustments. The monitoring will be carried out in accordance with the study plan and the procedures will be monitored continuously to ensure the research process.

### Plans for communicating important protocol amendments to relevant parties (e.g., trial participants, ethical committees) {25}

Any important protocol amendments will be communicated to the initiating study sponsor at first. The principal investigator will then notify the study centers. A copy of the revised protocol will be sent to the principal investigator and will be added to the Investigator Site File. Protocol modifications will be updated as they occur in ClinicalTrials.gov. Documentation will be provided to study sites for their local review and implementation as required. Furthermore, any deviations from the study protocol will be fully documented using a breach report form.

## Dissemination plans {31a}

This study will be published as an article in an international journal for peers to understand. Other interested researchers may consult the data of this experiment for further research.

## Discussion

This study is an interventional study to investigate the effects of target concentration of a single anesthetic exposure on postoperative sleep disturbance in female patients. The study also examines the potential of dexmedetomidine to reduce the incidence of POSD.

It is becoming increasingly evident that general anesthesia exerts a significant influence on postoperative sleep rhythm and/or circadian rhythm [18]. The incidence of postoperative sleep disturbance in patients undergoing general anesthesia and opioids is demonstrably higher than in patients undergoing regional anesthesia. Even in case of minor surgery or optimal postoperative analgesia, patients frequently experience sleep disturbance for up to 7 days following short-term exposure to propofol during endoscopy. Clinical research demonstrated that postoperative complications, such as postoperative fatigue syndrome, altered mental status, and cardiovascular accidents, are closely related to postoperative sleep disturbance [19, 20]. Consequently, improving sleep quality has a profound effect on postoperative recovery.

It has been demonstrated that surgical procedures under general anesthesia result in a significant phase shift in the diurnal sleep–wake rhythm and a deterioration in sleep quality [21]. Furthermore, it has been shown that traumatic surgical stress has a direct effect on the central nervous system, significantly altering sleep architecture and neurotransmitters. In addition, the role of anesthetics in inducing and exacerbating sleep disturbance has been suggested. To elucidate the clinical significance of anesthesia-related sleep disturbance, both biological investigations and clinical trials are necessary. It may also be important to develop a protocol that addresses the potential for sleep disturbance after general anesthesia. In animal studies, the majority of general anesthetics are either NMDA antagonists or GABA agonists that activate NMDA or GABA receptors in the suprachiasmatic nucleus [22]. This results in a shift of the diurnal rhythm, with the onset of melatonin secretion occurring at an earlier or later point in the day. Furthermore, observational studies in humans have also shown delayed melatonin secretion and glucocorticoid release [23]. The potential disruption of the circadian timing system resulting from sleep irregularity during anesthesia may have a negative impact on human health and may hinder an individual’s ability to recover from surgery [24]. Female patients are more likely to report emotional distress prior to surgery, and in our previous research, they were more likely to report sleep disturbance over the following 3 days. To enhance the precision of measurement, it is essential to examine the concentration-effect AUC of propofol in minor or non-apparent surgical procedures.

Hysteroscopic surgery is becoming increasingly popular in the twenty-first century due to its enhanced convenience and efficacy in women’s gynecological examinations and treatments. Given the brief anesthetic and surgical periods, and the minimally invasive nature of the procedure, both patients and physicians frequently fail to recognize the occurrence of postoperative sleep disorders. Previous studies indicated that women are at an increased risk of developing sleep disorders [5]. Interestingly, the study found that younger women undergoing hysterectomy were more likely to wake up during the night than older women [25]. Additionally, menopausal women exhibited poorer sleep trajectories than postmenopausal women [26]. Therefore, it is important to investigate the potential sleep issues associated with short-line hysteroscopy surgery in young and middle-aged women.

The administration of low-dose nocturnal dexmedetomidine may enhance postoperative sleep quality on the first night in the intensive care unit by maintaining forebrain connectivity. A small study found that daytime use of dexmedetomidine may be more effective than night-time use in improving postoperative sleep quality in patients aged 30 to 55 years undergoing laparoscopic surgery. These studies indicate that the use of dexmedetomidine may be a viable strategy for surgical patients [27]. If we can determine the effect of low-dose dexmedetomidine on propofol-associated sleep disturbance, we may not only improve postoperative recovery, but also ensure the optimal use of anesthetics in clinical practice.

## Trial status

Protocol version #3, June 15, 2020. Registered at ClinicalTrials.gov, ID: NCT06281561. Currently recruiting. The trial start date was April 2024 and will be completed in May 2025.

## Data Availability

The datasets used and/or analyzed after completing the current study will be available from the corresponding author by reasonable request.

## Abbreviations

TIVA: Total intravenous anesthesia
REM: Rapid eye movement
ASA: American Society of Anesthesiologists
DEX: Dexmedetomidine
BIS: Bispectral index
TCI: Target-controlled infusion
POD: Postoperative sleep disorder
NMDA: N-methyl-D-aspartate
GABA: Gamma-aminobutyric acid
AUC: Area under the plasma concentration–time curve
